# Fractalkine Mediates Communication between Pathogenic Proteins and Microglia: Implications of Anti-Inflammatory Treatments in Different Stages of Neurodegenerative Diseases

**DOI:** 10.1155/2012/345472

**Published:** 2012-08-05

**Authors:** Nicole M. Desforges, Michaeline L. Hebron, Norah K. Algarzae, Irina Lonskaya, Charbel E.-H. Moussa

**Affiliations:** Department of Neuroscience, Georgetown University Medical Center, Washington, DC 20057, USA

## Abstract

The role of inflammation in neurodegenerative diseases has been widely demonstrated. Intraneuronal protein accumulation may regulate microglial activity via the fractalkine (CX3CL1) signaling pathway that provides a mechanism through which neurons communicate with microglia. CX3CL1 levels fluctuate in different stages of neurodegenerative diseases and in various animal models, warranting further investigation of the mechanisms underlying microglial response to pathogenic proteins, including Tau, **β**-amyloid (A**β**), and **α**-synuclein. The temporal relationship between microglial activity and localization of pathogenic proteins (intra- versus extracellular) likely determines whether neuroinflammation mitigates or exacerbates disease progression. Evidence in transgenic models suggests a beneficial effect of microglial activity on clearance of proteins like A**β** and a detrimental effect on Tau modification, but the role of CX3CL1 signaling in **α**-synucleinopathies is less clear. Here we review the nature of fractalkine-mediated neuronmicroglia interaction, which has significant implications for the efficacy of anti-inflammatory treatments during different stages of neurodegenerative pathology. Specifically, it is likely that anti-inflammatory treatment in early stages of disease during intraneuronal accumulation of proteins could be beneficial, while anti-inflammatory treatment in later stages when proteins are secreted to the extracellular space could exacerbate disease progression.

## 1. Introduction

**Figure 1:**
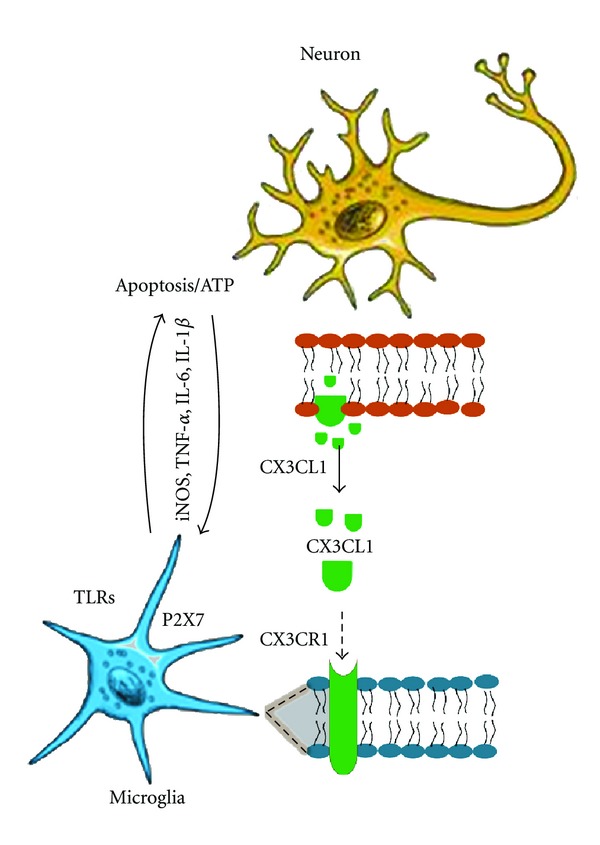
Initiation of inflammatory response following intraneuronal protein accumulation. Intraneuronal accumulation of pathogenic proteins causes ATP release by apoptotic neurons to activate purinergic microglia P2X7 receptors or TLRs. Activated microglia release proinflammatory cytokines (TNF-*α*, IL-16, IL-1) and iNOS to activate astrocytes (via MCP-1 chemotaxis) and increase apoptosis in stressed neurons. To initiate a neuroprotective immune response, injured neurons may communicate via fractalkine (CX3CR1) and suppress inflammation.

Increased microglial activity facilitates beneficial responses to central nervous system (CNS) injuries, including phagocytosis of debris and clearance of apoptotic cells; however, unregulated microglial activity can lead to production of neurotoxic factors that worsen CNS pathology and cause neuronal degeneration [1–7]. Microglia constitute the main immune cells in the CNS and provide innate immunity under physiological conditions and adaptive immunity under stress, promoting inflammation in response to various signals from apoptotic cells [1–4]. The phenotype of CNS resident macrophages is considered activated and designated M1 or “classical activation,” which describes the proinflammatory phenotypic response. M2 or “alternative activation” describes phenotypic responses to cytokines, such as Interleukin-(IL-) 4 and IL-13 [8]. In many neurodegenerative diseases, persistent injury (such as intraneuronal protein accumulation) promotes the production of proinflammatory molecules (Figure 1), like tumor necrosis factor (TNF)-*α*, Interleukin (IL)-1*β*, IL-6, reactive oxygen species (ROS), and nitric oxide (NO) [9]. Proinflammatory factors activate microglia [10, 11], which may remove not only apoptotic or damaged neurons, but also healthy neurons, aggravating the pathogenic process [5].

The inflammatory response is generally localized to areas of CNS injury via communication between immune cells and stressed neurons. Innate inflammation is reported in Alzheimer's disease (AD), Parkinson's disease (PD), and the Tauopathies (reviewed in [12]). In the healthy brain, microglia have a resting “deactivated” phenotype (ramified) [10]. Activated microglia are present in human postmortem brain tissues of patients with tauopathies, including AD, frontotemporal dementia with parkinsonism linked to chromosome-17 (FTDP), progressive supranuclear palsy (PSP), and corticobasal degeneration (CBD) [13–15]. Also associated with neurodegeneration in these diseases are hyperphosphorylated Tau (p-Tau) deposits [16–23]. It has been demonstrated in animal models of AD that the endotoxin lipopolysaccharide (LPS) promotes both inflammation and the accumulation of p-Tau [24] and that suppression of microglial activity prolongs survival in FTDP-associated P301L transgenic mice [25]. Our laboratory has previously shown a differential increase in microglial activity in response to accumulation of p-Tau in lentiviral wild type Tau versus mutant P301L mice at 1 month after-injection [26]. Cell culture models also demonstrate that proinflammatory cytokines can induce p-Tau [27–29]. These data suggest that microglial activity aggravates p-Tau through a common underlying mechanism moderating communication between microglia and neurons. Determining how this mechanism is temporally altered in response to p-Tau is critical to understanding the beneficial or detrimental role of microglial activity in different stages of disease pathology [30–32].

## 2. Fractalkine in Human Disease and Animal**** Models of Neurodegeneration

A central question in current research pertains to how communication between microglia and neurons, in which pathogenic proteins accumulate, affects the progression of inflammation. One inducer through which neurons and microglia can communicate to regulate inflammation is fractalkine (CX3CL1) (Figure 1). CX3CL1 is a 373-amino acid protein that has a chemokine domain located on top of a mucin-like stalk [33, 34]. Neurons secrete CX3CL1 [34], which exists in both membrane-bound and soluble forms [35]. The membrane-bound CX3CL1 can serve as an adhesion molecule for leukocytes expressing the fractalkine receptor (CX3CR1) [36] and soluble CX3CL1 can function as both a proinflammatory chemoattractant that activates receptive inflammatory cells [33, 37] and an anti-inflammatory [38], neuroprotective agent that reduces neuronal apoptosis [39]. The relationship between soluble CX3CL1 in peripheral blood and inflammatory diseases of the CNS is unclear. Several findings suggest that deletion of CX3CR1 increases microglial activity in various models of acute and chronic neuronal injury [40–43]. Fluctuations in CX3CL1 levels are also observed in many neurodegenerative diseases. Increased levels of serum CX3CL1 are reported in patients with multiple sclerosis [39, 44], traumatic brain injury [45], and human immunodeficiency virus (HIV) with CNS complications [46], but increased levels of serum CX3CL1 are not observed in patients with Guillain-Barré Syndrome and viral and bacterial meningitis [44]. Genetic variants with reduced levels of CX3CR1 are linked to age-related macular degeneration in humans [47].

CX3CL1 and its cognate receptor CX3CR1 may play an important role in immunoregulation in animal models of neurodegeneration. CX3CL1 expression is decreased in the cerebral cortex and hippocampus in the aged brains of amyloid precursor protein (APP) transgenic mice [48]. Decreased CX3CL1 levels are also observed in aged AD transgenic mouse models (Tg2576) in association with increased A*β* levels [48]. Microglial activity was increased while the levels of A*β* load and CX3CR1 were decreased in MyD88^−/−^ mice, suggesting CX3CL1 involvement in A*β* clearance [49]. CX3CR1 deficiency leads to decreased levels of A*β* deposition and protects against A*β* toxicity in transgenic mouse models of AD [50, 51]. LPS induces p-Tau of both endogenous and transgene-derived Tau in nontransgenic mice and in a humanized mouse model of Tauopathy, depending on LPS dose and CX3CR1 deficiency [40]. Additionally, impairment of CX3CL1 signaling pathway leads to deterioration in cognitive function and synaptic plasticity via alteration of IL-1*β* function [52]. Although CX3CR1 deficiency exacerbates AD-related neuronal and behavioral pathologies in mice overexpressing human A*β*, these effects are likely to be associated with the level of cytokine production and not A*β* plaque load, suggesting that alteration of proinflammatory factors, including TNF-*α* and IL-6 may modulate CX3CL1 signaling [43]. Conversely, production of NO, IL-6, and TNF-*α* may be inhibited by CX3CL1 [53, 54].

Exogenous CX3CL1 is neuroprotective in some other models of neuroinflammation [55, 56], and disruption of CX3CL1-CX3CR1 communication by deletion of the CX3CR1 gene causes neurotoxicity in mouse models of systemic inflammation, PD, and amyotrophic lateral sclerosis [57] but protects against neuronal loss in a mouse model of focal cerebral ischemia [58]. CX3CR1 knockout mice show more toxicity and substantia nigra (SN) degeneration in response to LPS treatment following administration of 1-methyl-4-phenyl-1,2,3,6-tetrahydropyridine (MPTP), a neurotoxic precursor of 1-methyl-4-phenylpyridinium (MPP+) [57]. Together, these studies suggest altered microglial activity through CX3CL1 signaling, which may play a direct role in immunoregulation depending upon the CNS insult. CX3CL1-CX3CR1 signaling is therefore a possible mediator of communication between injured neurons and microglia and may play a significant role in the regulation of microglial activity in response to pathogenic protein accumulation in early, or protein secretion, in later stages of disease.

## 3. Intraneuronal A*β* and Inflammation in Early**** Stages of AD

A primary feature of AD is the presence of extracellular aggregates of A*β* peptide (plaques) and intracellular inclusions (tangles) containing p-Tau [59–61]. Variants of A*β* peptide, including A*β*42 and A*β*40, are produced by the cleavage of APP and subsequent cleavage of an intermediate fragment, APP C-terminal fragments (CTFs) [62]. Cleavage of APP at an alternative site within the A*β* region by the cleaving enzyme *α*-secretase precludes A*β* formation [63, 64]. The causal association between mutations in APP and the onset of familial AD supports the role of A*β* in AD pathogenesis [65]. It is likely that in the early stages of disease, A*β* accumulates intraneuronally prior to the formation of extracellular plaques [66]. It is also likely that the intracellular pool of A*β* is externalized as neurons die, contributing to the formation of senile plaques [67–70]. The presence of intraneuronal A*β* is significant in that such a presence constitutes a preplaque stage of AD pathology. Our laboratory has previously shown that intraneuronal A*β* induces microglial and astrocyte activation and increases inflammatory markers in gene transfer models [71, 72]. Furthermore, it has been shown that intraneuronal A*β* can cause apoptosis and cell death, which stimulate microglial and astrocyte activation independently of extracellular plaques [73]. These results implicate communication between microglia and A*β* expressing neurons in the onset of inflammation in AD. Inflammation has been associated with neurodegenerative disease etiology in AD, in which A*β* and Tau can act as inflammatory stimuli to promote microglial activity [1, 74–76]. Therefore, inflammation in AD may arise not only from extracellular plaque formation, but also as a consequence of communication between microglia and A*β*-expressing neurons. Accumulation of intraneuronal A*β* can induce damage to lysosomes and multivesicular bodies, leading to leakage of A*β* from vesicles into the cytosol and activation of inflammatory mechanisms without extracellular accumulation of amyloid plaques. Several studies have suggested that manipulation of chemokines and/or their receptors may be a therapeutic target in neurodegenerative diseases, including AD [77–79]. Microglia treated with recombinant CX3CL1 or IL-34 partially protect against A*β* toxicity via enhancement of A*β* clearance and antioxidant production [80]. Significant differences in CX3CL1 levels were detected in a cohort of 51 patients with mild cognitive impairment (MCI), 51 AD patients and 57 controls [81]. However, the increase in plasma CX3CL1 levels is not congruent with tissue levels, which are decreased in the hippocampus and frontal cortex of advanced AD cases [43], suggesting variable roles of CX3CL1 in different stages of AD pathogenesis. The level of plasma soluble fractalkine was significantly higher in MCI and moderate AD patients compared to severe AD, suggesting that higher levels of soluble plasma fractalkine is associated with greater cognitive impairment [81]. Therefore, the fractalkine signaling pathway that mediates communication between microglia and neurons is deficient in AD brains and downregulated by A*β*.

## 4. Fractalkine in PD-Related Inflammation

The characteristics of PD include death of dopaminergic neurons in the SN [50, 58, 82] and formation of Lewy bodies (LBs) [83–92], or inclusions comprised mainly of *α*-synuclein [83–99]. The simultaneous occurrence of *α*-synuclein and Tau pathology is observed in multiple system atrophy (MSA), though the mechanisms underlying a possible connection between the two proteins are unknown [100, 101]. Early onset familial PD arises from mutations in the autosomal recessive genes PARKIN, PTEN-induced kinase-1 (PINK1), and DJ-1 [94] while late onset PD is associated with dominantly-inherited mutations in leucine-rich repeat kinase 2 (LRRK2) and *α*-synuclein.

Aggregation of *α*-synuclein is implicated in the activation of microglia and subsequent inflammation associated with PD. It was previously thought that *α*-synuclein-related pathology was confined to within neurons, but recent research suggests that microglia are activated following the release of *α*-synuclein aggregates into the extracellular space by apoptotic cells [102]. However, extracellular *α*-Synuclein has not been found in PD brains. Aggregated forms of *α*-synuclein induce microglial activation [99, 103]. Several microglia-derived inflammatory factors (ROS, NO, TNF-*α*, and IL-1*β*), as well as LPS, promote death of dopaminergic neurons [104–106]. The phagocytosis of *α*-synuclein by microglia induces NADPH oxidase activity and the production of ROS [103]. These neurotoxic effects signify a contributory role of microglia and inflammation in PD pathology. Inflammation has also been detected in PD brains lacking LBs, such as parkin-linked autosomal recessive early onset PD [9]. These cases, as well as the role of Tau as a risk factor for PD, suggest that additional mechanisms regulate inflammation. For example, CX3CL1 suppresses microglial activation and protects against neuronal loss and striatal lesion in 6-hydroxydopamine (6-OHDA) rat model of PD [107]. MPP+ increases neuronal CX3CL1 levels in rat SN, but administration of CX3CR1 antagonists blocks PD-like pathology, including loss of dopaminergic neurons and motor behavior [108], suggesting that fractalkine can modulate microglial activation in PD models. Deletion of CX3CR1 aggravates microglial neurotoxicity in response to LPS in the MPTP model of PD and in the superoxide dismutase 1 (SOD1) G93A model of ALS [57], suggesting that CX3CL1 signaling may limit microglial toxicity [57]. The level of plasma soluble CX3CL1 also correlates positively with disease severity and progression in human PD patients, suggesting that CX3CL1 can be used as a biomarker to differentiate between neurodegenerative diseases [109].

## 5. The Effects of Microglial Activation Depend on Disease Stage

Whether inflammation rescues or exacerbates cell death in neurodegenerative disease likely depends on the stage of disease progression. Microglial activation facilitates the removal of apoptotic cells and toxins from the CNS, releasing neurotrophic factors that aid in repair following injury [5]. However, microglia also release inflammatory markers that can induce apoptosis. The apparent ambivalence of increased microglial activity is associated with unsuccessful attempts to provide anti-inflammatory treatment in human clinical trials. Preliminary clinical trials in which nonsteroidal anti-inflammatory drugs (NSAIDs) were administered before the development of neurodegeneration suggested that disease risk was reduced by inhibition of the immune response [110, 111]. However, later trials found that anti-inflammatory drugs were harmful in AD patients [110]. These conflicting data reflect the current lack of understanding of the role of the immune response in CNS diseases and point to the importance of the temporal relationship between the disease stage and the anti-inflammatory intervention.

The timing of the immune response in relation to disease progression complicates the use of anti-inflammatory treatment in various CNS diseases. For example, the permanence of brain damage following stroke or ischemia depends on the activity of proinflammatory cytokines, the activation of microglia, and the recruitment of leukocytes [112, 113]. It has been found that inhibiting TNF-*α* and IL-1, which mediate postischemic activity by attracting leukocytes to the injury or by damaging cells directly, confers neuroprotection in animal models of stroke [112, 113]. In AD models, the involvement of innate immunity via microglial activation and phagocytosis of A*β* renders anti-inflammatory therapy particularly relevant to the study of AD [114–116]. In AD patients, however, deficits in Toll-like receptors (TLRs) expression inhibit the removal of A*β* from the brain and result in lack of A*β* clearance by macrophages [117], and TLR2 deficiency in AD mouse models is associated with severe cognitive impairment [118]. In addition to anti-inflammatory treatment, intervention in the hematopoietic system has been suggested as a possible model of treatment for AD. The administration of macrophage colony-stimulating factor, a hematopoietic cytokine, to mouse microglia promotes degradation of internalized A*β* *in vitro* [119] and protects against cognitive decline *in vivo* when administered prior to the development of learning and memory deficits [120], supporting the importance of timing of anti-inflammatory treatment relative to disease progression. Taken together, these findings support the targeting of innate immune cells as a therapeutic approach for AD and other neurodegenerative diseases. However, conflicting data from clinical trials necessitate further investigation of the role of the immune response in disease development and progression.

## 6. Putting It into Perspective

**Figure 2:**
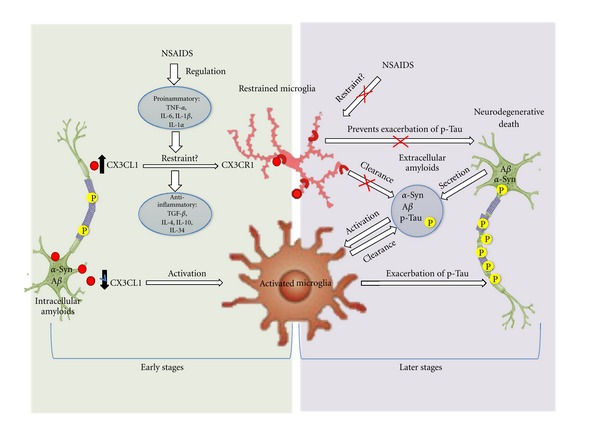
Modulation of CX3CL1 in early versus late disease stages. The success of anti-inflammatory treatment in neurodegenerative diseases likely depends on the stage of disease progression. Treatment with NSAIDS early in disease pathology may alter the levels of various proinflammatory markers, including TNF-*α*, IL-6, IL-1*β*, and IL-1*α*, and anti-inflammatory markers, including TGF-*β*, IL-4, IL-10, and IL-34. The changes in the levels of these cytokines may lead to altered CX3CL1 signaling, which would either increase microglial activity (if CX3CL1 were reduced) or restrain microglia (if CX3CL1 levels were increased). In later stages of disease, secretion of pathogenic proteins like A*β*, *α*-synuclein, and p-Tau to the extracellular space increases microglial activation. Microglial activity promotes p-Tau, which destabilizes microtubules and leads to cell death. Treatment with NSAIDS in later stages of disease would likely be detrimental, as restraining microglia would weaken the immune response to remove extracellular protein aggregates.

 Research on the suppression of microglial activity has been actively pursued with limited success [121] and strategies to manipulate the protective role of microglia—the detection and removal of apoptotic cells—have not been fully investigated [122–124]. These strategies warrant further research, as apoptotic cells that enter secondary necrosis [125] and trigger inflammation [126, 127] increase tissue damage. In this context, the role of CX3CL1 in mediating communication between preapoptotic neurons and microglia becomes greatly important. Such intervention would be relevant in early stages of disease progression, during which intracellular accumulation of pathogenic proteins anticipates apoptosis and the formation of extracellular protein aggregates. In later stages of disease pathology, decreased CX3CL1 signaling may activate microglia and induce p-Tau (Figure 2), which exacerbates disease progression by promoting apoptosis. Additionally, the use of NSAIDs to restrain microglial activity may exacerbate pathology due to lack of phagocytic clearance of secreted extracellular amyloids, including *α*-Synuclein, A*β* and p-Tau. In this context, targeting microglial activity in later disease stages may be detrimental and contributory to disease progression. However, targeting the CX3CL1 pathway in early disease stages could be beneficial, at least in delaying disease progression via restraint of microglial activity. Along this line of thought, NSAIDs administration could regulate key proinflammatory cytokines (Figure 2) that would modulate CX3CL1 signaling and microglial activity. It remains to be fully elucidated when and how alteration of proinflammatory markers may increase or decrease CX3CL1 signaling, which may either activate or suppress microglia. One possibility is increased CX3CL1 levels to restrain microglial activity and prevent the exacerbation of p-Tau damage. However, this intervention should be timed to avoid interference with microglial activity when patients progress into more advanced stages of disease, during which removal of extracellular deposits becomes necessary. Therefore, understanding the critical interplay between proinflammatory (TNF-*α*, IL-6, IL-1*β*, and IL-1*α*), anti-inflammatory cytokines (IL-10, TGF-*β*, IL-34, etc.), and fractalkine levels to modulate microglial activity is highly significant. Furthermore, whether the activation of microglia in the context of neurodegenerative disease is beneficial or detrimental may also depend upon the type of disease. Successful anti-inflammatory treatments of CNS diseases will likely be specific not only to the stage of disease pathology, but also to the type of disease. It has been found that many of the same cytokines are implicated in the pathology of AD, PD, and ALS despite distinct patterns of neuronal loss in each disease [9]. Previous literature presents contradictory evidence regarding the effects of targeting microglia in various CNS diseases. Glass et al. [128], for example, suggest that targeting microglia in PD and ALS is detrimental while other studies suggest that targeting microglia aids in A*β* clearance in AD. Further investigation of the role of the inflammatory response in each disease will determine the potential for anti-inflammatory treatments. Here we suggest a temporally-defined strategy of intervention in which early targeting of CX3CL1 signaling slows disease progression and prevents p-Tau formation.
